# A comprehensive analysis of metabolomics and transcriptomics in non-small cell lung cancer

**DOI:** 10.1371/journal.pone.0232272

**Published:** 2020-05-06

**Authors:** Chen Ruiying, Li Zeyun, Yuan Yongliang, Zhu Zijia, Zhang Ji, Tian Xin, Zhang Xiaojian

**Affiliations:** 1 Department of Respiratory Medicine, The First Affiliated Hospital of Zhengzhou University, Zhengzhou, china; 2 Department of Pharmacy, The First Affiliated Hospital of Zhengzhou University, Zhengzhou, china; 3 Henan Key Laboratory of Precision Clinical Pharmacy, Zhengzhou University, Zhengzhou, china; Baylor College of Medicine, UNITED STATES

## Abstract

Non-small cell lung cancer (NSCLC) remains a leading cause of cancer death globally. More accurate and reliable diagnostic methods/biomarkers are urgently needed. Joint application of metabolomics and transcriptomics technologies possesses the high efficiency of identifying key metabolic pathways and functional genes in lung cancer patients. In this study, we performed an untargeted metabolomics analysis of 142 NSCLC patients and 159 healthy controls; 35 identified metabolites were significantly different between NSCLC patients and healthy controls, of which 6 metabolites (hypoxanthine, inosine, L-tryptophan, indoleacrylic acid, acyl-carnitine C10:1, and lysoPC(18:2)) were chosen as combinational potential biomarkers for NSCLC. The area under the curve (AUC) value, sensitivity (SE), and specificity (SP) of these six biomarkers were 0.99, 0.98, and 0.99, respectively. Potential diagnostic implications of the metabolic characteristics in NSCLC was studied. The metabolomics results were further verified by transcriptomics analysis of 1027 NSCLC patients and 108 adjacent peritumoral tissues from TCGA database. This analysis identified 2202 genes with significantly different expressions in cancer cells compared to normal controls, which in turn defined pathways implicated in the metabolism of the compounds revealed by metabolomics analysis. We built a fully connected network of metabolites and genes, which shows a good correspondence between the transcriptome analysis and the metabolites selected for diagnosis. In conclusion, this work provides evidence that the metabolic biomarkers identified may be used for NSCLC diagnosis and screening. Comprehensive analysis of metabolomics and transcriptomics data offered a validated and comprehensive understanding of metabolism in NSCLC.

## 1. Introduction

Non-small cell lung cancer (NSCLC) is the leading cause of cancer death globally. The incidence and mortality rates of lung cancer have significantly increased in recent years [1]. NSCLC is generally diagnosed by computed tomography (CT) and followed by percutaneous lung biopsy. However, early diagnosis using non-invasive and cost-effective methods attracts increasing attention. To develop better diagnostics and more effective treatments, research in the past decades has focused on the identification of molecular changes in the genome, transcriptome, proteome, and more recently also in the metabolome [2].

Metabolomics, currently the predominant methodology for early diagnosis and precision medicine, focuses on small molecules to reveal alterations in the metabolism of biological systems. Metabolomics could also gain insight into the progression of cancers from a metabolic view [3]. The metabolomics techniques commonly employed in lung cancer diagnosis are nuclear magnetic resonance analysis (NMR), gas chromatography/mass spectrometry (GC/MS), and liquid chromatography/mass spectrometry (LC/MS) [4]. Among these techniques, LC/MS is the most frequently used, because it has the advantages of high sensitivity and high throughput. Metabolite profiling is a relatively underrepresented field of biomarker development for identifying and characterizing lung cancer, revealing differences in concentration of metabolites or alterations of metabolic pathways. Previous studies have identified metabolite differences in lung cancer patients related to altered amino acid metabolism, glycolysis and gluconeogenesis, protein metabolism, handling of oxidative stresses, and fatty acid metabolism [3–9]. NSCLC is a heterogeneous disease covering a heterogeneous population, with a complex system for classifying the disease state and progression [10]. However, the sample sizes employed in previous work were limited, and the validation of larger numbers of samples is needed to provide more reliable information.

Transcriptomics enables us to interpret the functional elements of the genome and reveal the global gene expression profiles associated with the disease. Integration of metabolomics with transcriptomics data has recently been used in cancer research and may lead to more insight into these fields than either approach alone. Finding that dysregulation of metabolites and genes occur in the same biological processes in cancer provides enhanced validation to potential diagnostic biomarkers.

**Fig 1:**
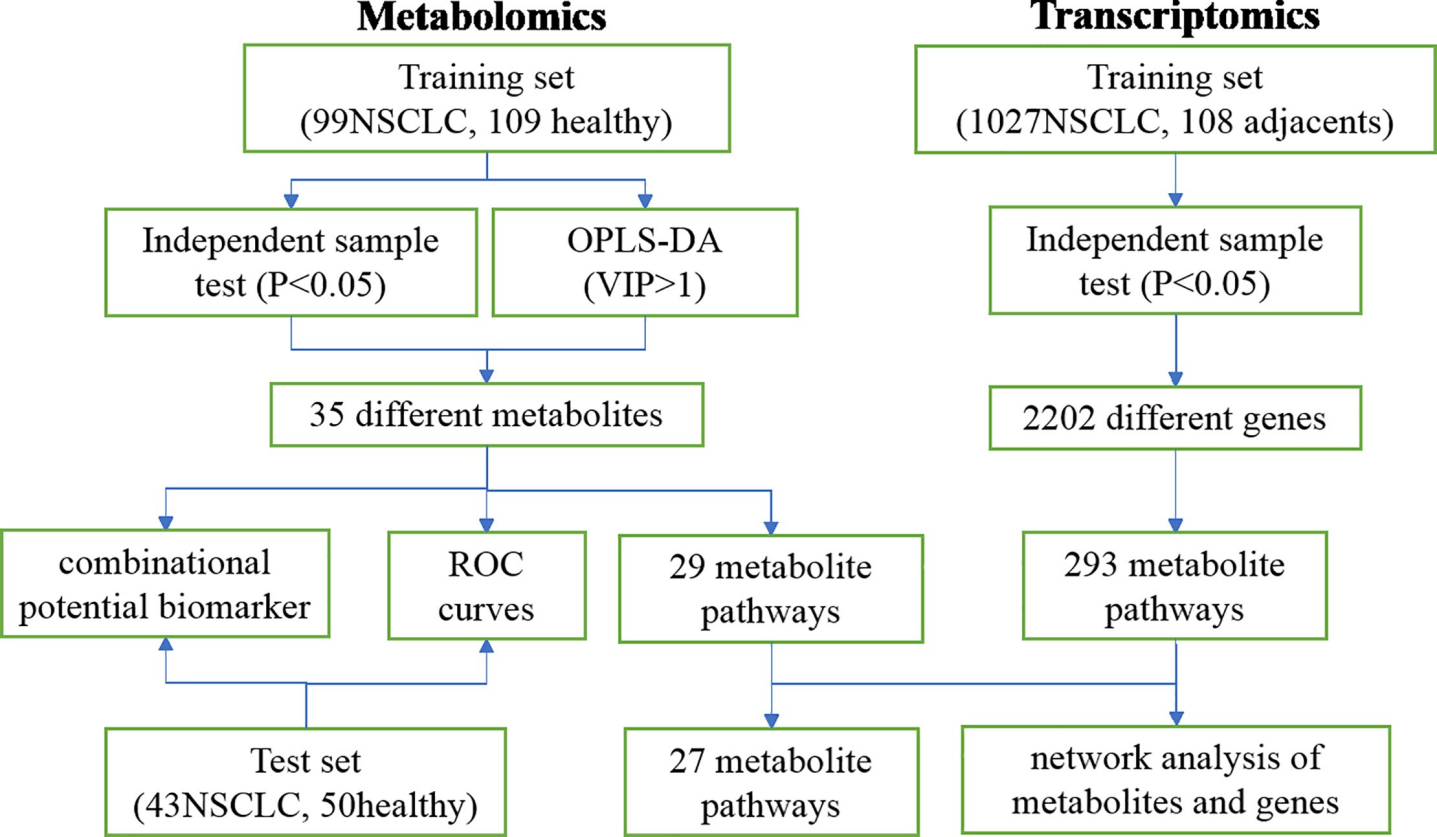
An overview workflow of the comprehensive analysis of metabolomics and transcriptomics in NSCLC.

To date, no study has been conducted to explore NSCLC through the integration of metabolomics and transcriptomics with large sample sizes. Here, we carried out a comprehensive analysis of metabolomics and transcriptomics data to discover dysregulated pathways and identify more reliable biomarkers for the diagnosis of NSCLC. We performed serum metabolomic analysis of samples from patients with NSCLC and healthy controls using ultra-high-performance liquid chromatography/quadrupole time-of-flight mass spectrometry (UPLC/Q-TOF MS). In addition, we conducted transcriptomics analysis of data from the cancer genome atlas (TCGA, https://www.cancer.gov/about-nci/organization/ccg/research/structural-genomics/tcga/?redirect=true) database to validate dysregulated pathways and to clarify relationships between the mis-regulation of genes and metabolites. The overview workflow of the comprehensive analysis of metabolomics and transcriptomics in NSCLC is summarized in Fig 1. The integration of metabolomics and transcriptomics analysis will produce more reliable biomarkers and provide a complementary method for cancer detection.

## 2. Experimental

### 2.1. Chemicals and reagents

We purchased acetonitrile and methanol (HPLC grade) from Fisher Scientific (Fairlawn, NJ, USA). Formic acid (HPLC grade) was from Fisher Scientific (Waltham, MA, USA). Ammonium acetate (HPLC grade) was purchased from Beijing Reagent Company (Beijing, China). Distilled water was filtered through the Milli-Q system (Millipore, MA, USA).

### 2.2. Metabolomics sample collection and preparation

All serum samples were collected from fasting subjects and provided by the First Affiliated Hospital of Zhengzhou University, Zhengzhou, China from November 2011 to July 2018. The study was approved by the Institutional Review Board of the First Affiliated Hospital of Zhengzhou University. All subjects provided written informed consent according to the institutional guidelines. After collection, all serum samples were kept at -80°C until use.

The inclusion criteria of patients were as follows: patients were newly diagnosed with NSCLC and had not received previous surgery, radiotherapy, or chemotherapy. The cancer diagnosis was performed by histopathological analysis of tissue specimens. Healthy volunteers, none of whom had known chronic or major diseases, or were undergoing any treatment, were matched for weight and sex with the patients enrolled. We collected one batch of serum samples from 99 NSCLC patients (31 with squamous carcinoma and 68 with adenocarcinoma) and 109 healthy controls at the discovery phase. The age and sex information are provided in Table 1. We used another batch of serum samples from 43 NSCLC patients (18 with squamous carcinoma and 25 with adenocarcinoma) and 50 healthy controls at the validation phase. The two batches were treated with the same preparative and analytic methods.

**Table 1:** Characteristics of the metabolomics study population.

	Training set(n = 208)			validation set(n = 93)		
	NSCLC	Healthy	*p*^a^	NSCLC	Healthy	*p*^a^
**number**	99	109		43	50	
**Anthropometric characteristics**						
**age (years)**	55.5 (39,67)	44 (35, 69)	<0.0001	57 (38,68)	42 (33,55)	<0.0001
**gender (M/F)**	54/45	64/45	0.544	27/15	24/26	0.117
**Weight (kg)**	66.5(45, 88)	77.1(44.6, 88.3)	0.1002	69(45,86)	64.7(46,87)	0.218
**Cancer type**						
**Squamous carcinoma**	31			18		
**Adenocarcinoma**	68			25		

^a^Unpaired t tests for continuous measures and χ2 tests for categorical variables

Small molecule metabolites were extracted from serum after removing macromolecules by the addition of 400 μL acetonitrile to 100 μL of serum, vortexing for 1 min, and centrifugation at 14,000 rpm for 10 min at 4°C. Next, two 200 μL aliquots of supernatant were lyophilized for analysis in positive and negative electrospray ionization (ESI+ and ESI-) mode. The lyophilized samples were re-dissolved in 100 μL acetonitrile/water (20:80, v/v) for injection.

QC samples were prepared by combining equal aliquots from all serum samples and were injected every five specimens during the whole analysis. The QCs were used to test the instruments, equilibrate the UPLC/Q-TOF MS system before sample injection, and examine the stability of the system during the analytical procedure. All samples were prepared and analyzed from 11 December 2018 to 17 December 2018.

### 2.3. UPLC/Q-TOF MS based metabolome profiling

The sample analysis was performed on a UPLC-ESI-Q-TOF system (AB Sciex, Framingham, MA, USA). Chromatography separation was achieved on an ACQUITY UPLC HSS T3 column (100 × 2.1 mm i.d., 1.8 μm, Waters Co., MA, USA) maintained at 30°C in both ESI+ and ESI- mode. For ESI+, the mobile phase consisted of A (0.1% formic acid in water) and B (0.1% formic acid in acetonitrile); for ESI-, the mobile phase consisted of C (5 mM ammonium acetate in water) and D (5 mM ammonium acetate in 10% water/acetonitrile). Gradient elution was carried out using 0–1 min, 5–30% B or D; 1–3 min, 30–60% B or D; 3–10 min, 60–95% B or D; 10–12 min, 95–95% B or D; 12–12.1 min, 5% B or D; and 12.1−14 min, 5% B or D in ESI+ or ESI- mode. The flow rate was set at 0.4 mL/min, and the injection volume was 3 μL in both ESI+ and ESI- modes.

The mass spectrometric data were collected on a SCIEX X500R QTOF mass spectrometer (AB Sciex, Framingham, MA, USA) coupled with an electrospray ionization interface (ESI). We employed SCIEX OS software 1.2 (AB, Milford, MA, USA) for data acquisition and processing. The electrospray ion source temperature and spray voltage were set to 600°C, 5500 V, and 600°C, -4500 V, for the ESI+ and ESI- modes, respectively. The declustering potential (DP), collision energy (CE), Collision Energy Spread (CES), gas 1, gas 2, and curtain gas were 80 V, 40 V, ±20 V, 60 psi, 60 psi, and 35 psi, respectively. Nitrogen was kept as the nebulizer and auxiliary gas. TOF MS and TOF MS/MS were scanned with the mass range of m/z 50–1000. Continuous recalibration was carried out after every sixth sample. Also, dynamic background subtraction (DBS) trigger information-dependent acquisition (IDA) was used to obtain MS/MS data for low-level constituents. The accurate mass and composition for the precursor ions and fragment ions were analyzed using the Markerview^™^ software (Version 4.1, Waters Co., Milford, MA, USA) integrated with the instrument.

### 2.4. Metabolomics data analysis

Raw data from TOF-MS were analyzed using Markerview for peak deconvolution and peak alignment with the following parameters: initial retention time, 0.5 min; final retention time, 23 min; mass tolerance, 20 PPM; ion intensity threshold, 300 counts; and retention time tolerance, 0.1 min. The data were combined into a single matrix by aligning peaks with the same mass-retention time features from each data file in the dataset. According to the 80% rule, the background and biologically irrelevant information were eliminated, and only variables with values above zero presenting in at least 80% of each group were kept for the following analysis. The ion intensities of each peak detected (2241 MS features for the ESI+ mode and 666 for the ESI- mode) were normalized to the sum of the peak intensities in each sample.

We imported output data from TOF-MS separately into SIMCA (version 14.0, Umetrics, Umeå, Sweden) for multivariable analysis (unit variance scaled). To provide comparative interpretations and visualization of the metabolic differences between NSCLC patients and healthy controls, principal component analysis (PCA) and orthogonal signal correction partial least-squares discriminant analysis (OPLS-DA) were applied. We describe the quality of the models by R2X and Q2 values. R2X shows the proportion of variance in the data explained by the models and indicates the goodness of fit. The closeness of a value to 1 indicates the goodness of fit. Q2, in contrast, shows the proportion of variance in the data predictable by the model and indicates predictability. The results are visualized in the form of score plots, where each point represents an individual sample (to show the group clusters), and loading plots or S-plots, where each coordinate represents one mass-retention feature (to identify the variables contributing to the classification). The variable importance of projection (VIP) is the vector that summarizes the total importance of the variable in explaining the model. The corresponding variables with VIP >1.0 were chosen as potential discriminant metabolites. Statistical analysis was also performed using a one-way analysis of variance (ANOVA) followed by Tukey’s multiple comparison test (SPSS, Chicago, IL, USA). Metabolite with P < 0.05 was considered to be statistically significant between two groups.

LC-MS Peaks were identified according to actual mass, MS/MS fragments, and retention time (RT). First, the m/z value of the molecular ion of interest was searched against an in-house human metabolites database, where data were collected from published research works. Then, the putative identifications were verified by comparing the MS2 fragmentations from the human metabolome database (HMDB) and Metabolite HR-MS/MS library (v1.0, AB, Milford, MA, USA).

Potential metabolite biomarkers that can distinguish NSCLC from healthy controls were randomly screened using a binary logistic regression model. Receiver operating characteristic (ROC) analysis was performed. Variables with AUC (area under the curve) values larger than 0.8 were identified as potential metabolite biomarkers and were further validated at the validation phase of 43 NSCLC patients and 50 healthy controls.

### 2.5. Transcriptomics analysis

The joint application of the metabolomics and transcriptomics possesses the high efficiency of identifying key metabolic pathways and functional genes in lung cancer. Although transcriptomics data was not acquired from serum samples collected, the transcriptomics data from the TCGA database shall verify result revealed by metabolomics analysis and prompt understanding of metabolic pathways interrupted. Thus, transcriptomics analysis from the TCGA database comprising 1027 samples of cancerous tissue and 108 samples of adjacent peritumoral tissue were conducted (Project ID TCGA-LUAD, TCGA-LUSC). Statistical testing was conducted using R version 3.4.0 as well as a modular open-source programming suite (https://www.r-project.org). Differentially expressed genes (DEGs) were selected by using the “DESeq. 2” package and analyzed through Student’s *t* test. Statistical significance was defined as P < 0.05 and fold change (FC) > 10.

## 3. Results and discussion

### 3.1. Serum metabolic profiling

**Fig 2:**
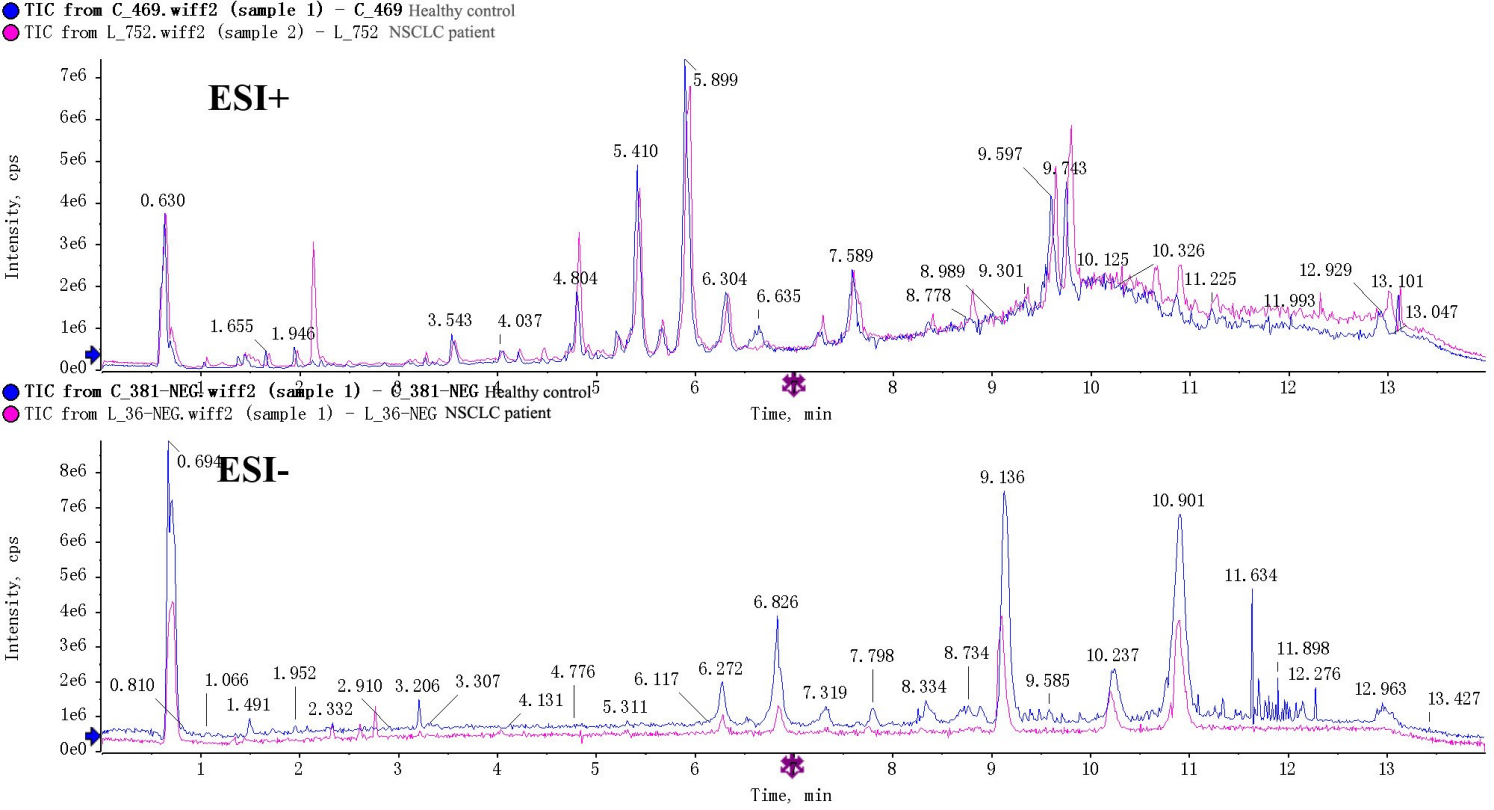
Representative TIC chromatograms of serum from NSCLC patient (red) and healthy people (blue) in ESI+ or ESI- mode.

Typical total ion current (TIC) chromatograms of serum metabolic profiles analyzed using UPLC/Q-TOF MS in the positive mode (ESI+) or negative mode (ESI-) are shown in Fig 2. No obvious differences between the NSCLC and healthy groups were detected by visual inspection. Original data in ESI+ or ESI- mode were imported into Markerview to produce data matrix with mass-feature and sample name as row and column titles. After eliminating zero values using the “80% rule,” 988 peaks from the ESI+ mode and 436 peaks from the ESI- mode were obtained for subsequent data analysis.

We used the QC samples to evaluate system stability and data quality. We found from the PCA score plots in both the positive and negative modes that all QC samples clustered together (S1 Fig, S1 Table), demonstrating the stability of the analytical system and the reliability of the data. Thus, the metabolic profiling data were reliable for classification and further analysis.

### 3.2. Multivariable analysis

Initially, PCA analysis after unit variance (UV) scaling was conducted to obtain a comprehensive and complete understanding of the metabolic profiling data. The PCA score plots of the patients with NSCLC and the healthy controls showed clear separation in both positive and negative mode (see S1 Fig).

**Fig 3:**
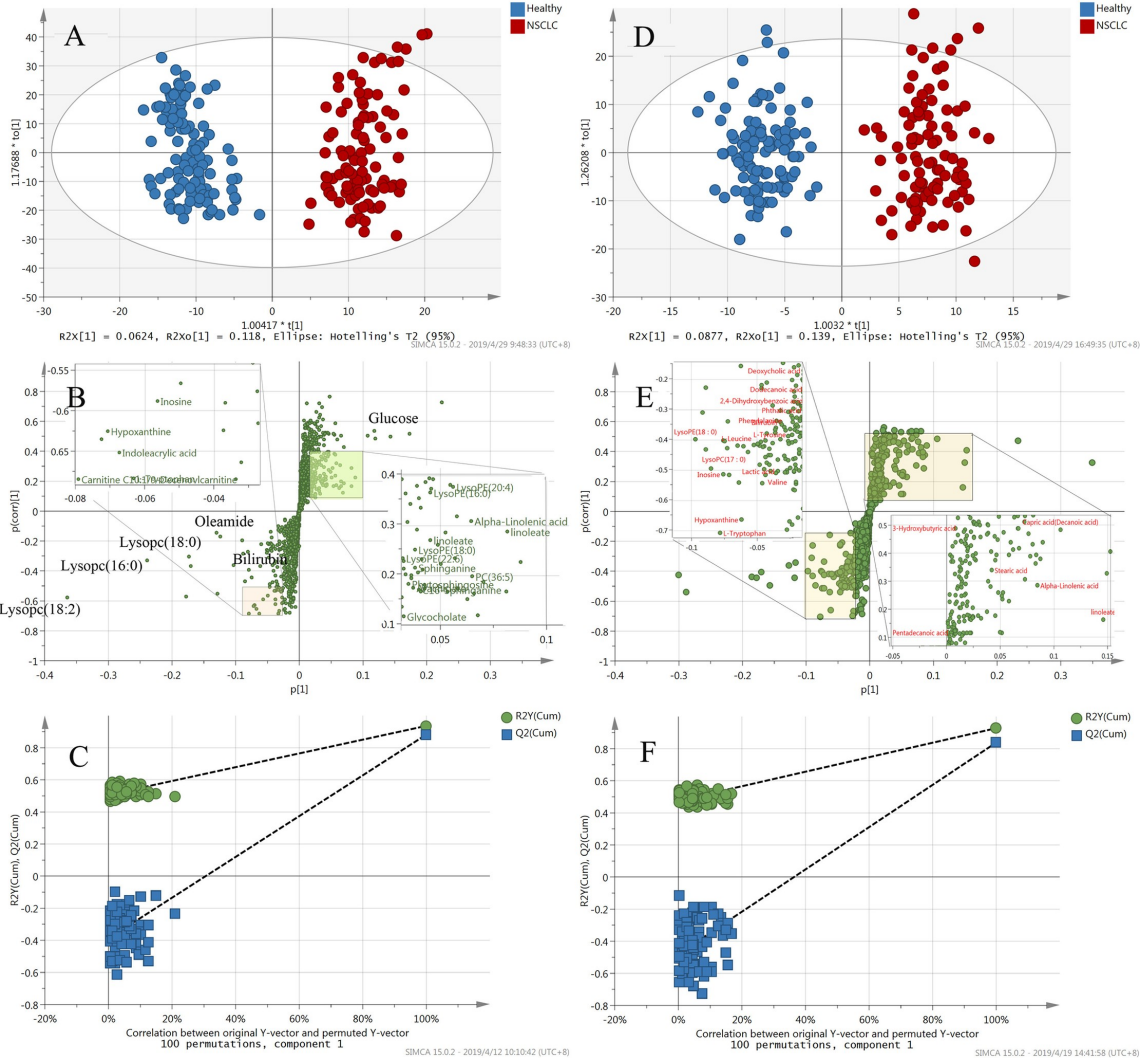
OPLS-DA score plots, S-plots, and validation plots for the metabolic profiling results of NSCLC patients and healthy people. (A) OPLS-DA score plot for NSCLC patients versus healthy controls in the ESI+ mode (R2X = 0.370, R2Y = 0.915, Q2 = 0.855). (B) OPLS-DA score plot for NSCLC patients versus healthy controls in the ESI- mode (R2X = 0.369, R2Y = 0.904, Q2 = 0.816). (C) S-plot for NSCLC patients versus healthy controls in the ESI+ mode. (D) S-plot for NSCLC patients versus healthy controls in the ESI- mode. (E) Permutation test for NSCLC patients versus healthy controls in the ESI+ mode. (F) Permutation test for NSCLC patients versus healthy controls in the ESI- mode. The criteria for stability and credibility are as follows: all permuted R2 and Q2 values on the left are lower than the original point on the right, and the Q2 regression line in blue has a negative intercept.

To further identify metabolites contributing to the separation of patients and controls, we established multivariable analysis models based on Pareto Variance (Par) scaling. In the ESI+ mode, an OPLS-DA model was developed, yielding a clear separation between the two groups (Fig 3A), with R2Y = 0.902 and Q2 = 0.867. In the ESI- mode, a similar result was obtained. The score plot (Fig 3B) showed a distinct separation of the patient group from the healthy group, achieving content modeling and predictive abilities (R2Y = 0.877 and Q2 = 0.828). In the S-plots (Fig 3C and 3D), the variables far from the center of the plot were assumed to contribute more to the model classification. No over-fitting for positive or negative mode was noticed according to the permutation validation (Fig 3E or 3F).

According to VIP list of S-plot, variables with VIP value over 1.0 were considered better correlated with the separation, and Student’s *t* test of these variables between the NSCLC and the healthy groups were conducted. We added the variables to the candidate list whose levels were statistically significant with P values below 0.05. The candidate list was searched against an in-house human metabolites database, HMDB as well as Metabolite HR-MS/MS library (v1.0, AB, Milford, MA, USA).

After these steps, we identified 35 significantly different variables in ESI+ or ESI- mode (S2 Table). The levels of inosine, hypoxanthine, valine, tyrosine, phenylalanine, and lysoPC decreased in the serum of NSCLC group compared to healthy controls, while those of arachidate, sphinganine, lysoPE (18:0), oleic acid, stearic acid, 3-hydroxybutyric acid, and capric acid were increased.

### 3.3. Screening of potential biomarkers

Potential metabolite biomarkers that can distinguish NSCLC from healthy controls are very valuable to auxiliary diagnosis and clinical applications. We used the metabolomic data to build separate linear classifier models that would distinguish the NSCLC from the healthy group. We used ROC analysis to assess the performance of the classifier models for group classification. In this study, all 35 differential metabolites of NSCLC were randomly screened. Results showed that the ROC curves of hypoxanthine, inosine, L-tryptophan, indoleacrylic acid, acyl-carnitine C10:1, and LysoPC (18:2) had AUC values larger than 0.8 (seen in Table 2). Particularly, hypoxanthine had the highest AUC value of 0.943, with 90.1% sensitivity and 94.1% specificity, and 95% confidence interval (CI) was [0.916, 0.970].

**Table 2:** AUC, SE, and SP of six biomarkers and the combination of these biomarkers for the training set and test set data.

Biomarkers	Training set			Test set		
	AUC	SE	SP	AUC	SE	SP
Hypoxanthine	0.943	0.901	0.941	0.935	0.900	0.900
Inosine	0.925	0.826	0.905	0.921	0.860	0.883
L-Tryptophan	0.870	0.733	0.882	0.791	0.780	0.717
Indoleacrylic acid	0.883	0.814	0.854	0.836	0.720	0.883
Carnitine C10:1	0.924	0.876	0.886	0.890	0.900	0.850
LysoPC(18:2)	0.821	0.795	0.727	0.813	0.800	0.733
Combined	0.975	0.957	0.950	0.957	0.960	0.900

AUC: area under the ROC curves; SE: sensitivity; and SP: specificity

**Fig 4:**
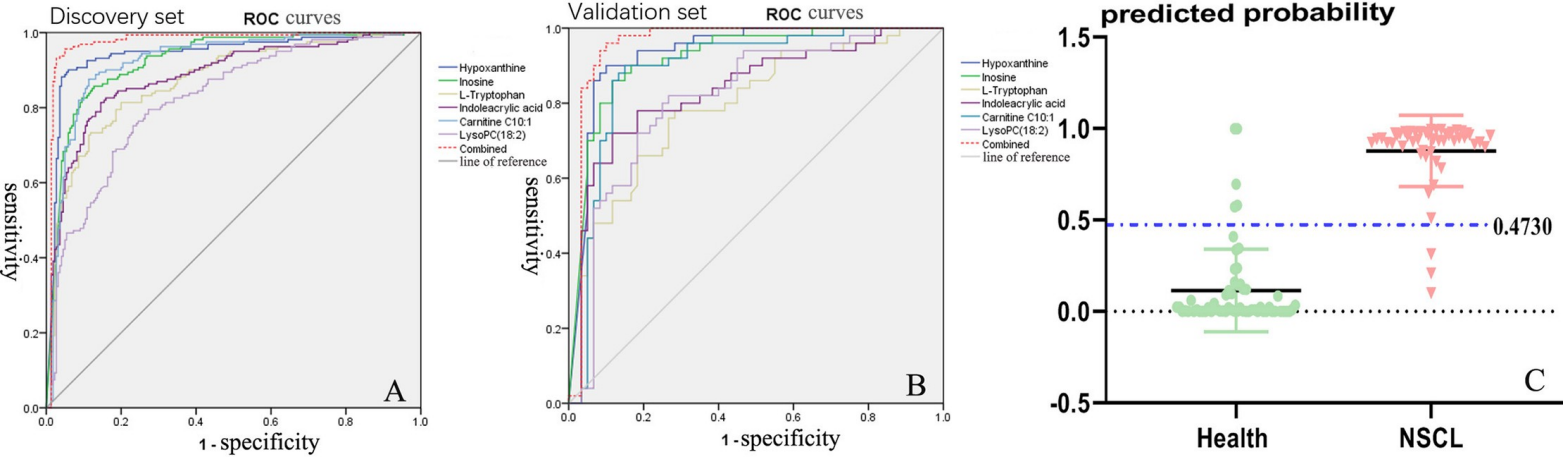
ROC curves of potential metabolic biomarkers for the discovery set (A) and validation set (B); Y-predicted scatter plot (C) of the validation set.

NSCLC is a complex disease involving changes in the levels of numerous metabolites in various metabolic pathways, so the combinational biomarker, which consists of two or more metabolites, could reflect the pathologic status of diseases more comprehensively. Sensitivity and specificity of the combinational biomarkers were additionally evaluated with ROC curves. It was found that combinational biomarkers achieved an AUC value of 0.975, while the statistical analysis provided 95.7% sensitivity and 95.0% specificity for the prediction of NSCLC and healthy control, with a cutoff value of 0.473. ROC curves are shown in Fig 4A.

Based on ROC analysis, six metabolites that had AUC values above 0.8 were chosen as combination metabolic biomarkers for NSCLC clinical diagnosis. (A): Combinational biomarkers achieved an AUC value of 0.975 for a discovery set of 99 cases of NSCL and 109 healthy controls. (B): Combinational biomarkers achieved an AUC value of 0.957 for a validation set of 43 cases of NSCLC and 50 healthy controls. (C): Y-predicted scatter plot of validation set (43 NSCLC cases and 50 healthy controls) predicted at a Y cutoff of 0.473; AUC, area under the curve; CI, confidence interval; LC, lung cancer; and ROC, receiver operating characteristic.

### 3.4. Validation of the combinational metabolic biomarker

To validate the combinational metabolic biomarker, we collected and analyzed another batch of serum samples from 43 NSCLC patients and 50 healthy controls using the above method. The validation results of combinational biomarkers are shown in Fig 4. The concentrations of hypoxanthine, inosine, L-tryptophan, indoleacrylic acid, acyl-carnitine C10:1, and lysoPC (18:2) were significantly decreased (P < 0.001), supporting the results from the previous sample batch. A ROC curve was generated to test the combinational potential biomarkers, yielding an AUC value was 0.957 (Fig 4B or Table 2), which confirmed that the sensitivity and specificity were satisfactory. Moreover, the combinational markers could evidently distinguish the NSCLC group from the healthy group (Fig 4C). Thus, the prospective combinational biomarkers appeared to have the potential to provide an auxiliary diagnosis of NSCLC. A large number of samples were required to confirm this conjecture.

### 3.5. Metabolomics pathway analysis

**Fig 5:**
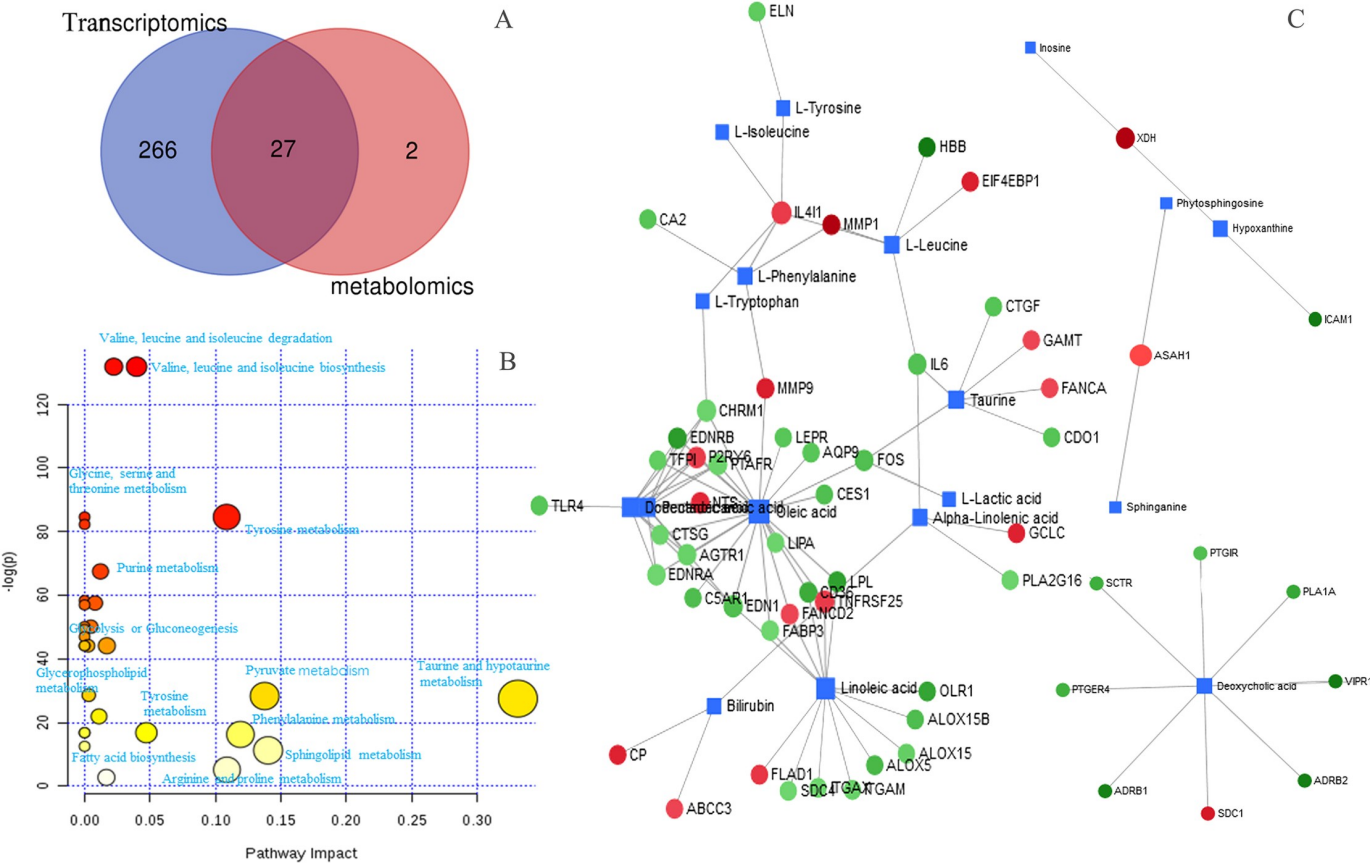
Venn plot (A) of metabolic pathways enriched by metabolomics and transcriptomics; pathway analysis plot (B) of metabolites with a significant difference between samples from NSCL patients and healthy controls; connected network (C) of metabolites and genes of metabolomics and transcriptomics analysis. The quadrangle in blue indicates differential metabolites, and the nodes in green or red indicate increased or decreased expression of genes.

The 35 differential metabolites between samples from NSCLC patients and normal controls were used for pathway analysis conducted using MetaboAnalyst 4.0 (http://www.metaboanalyst.ca). A total of 29 pathways were enriched (Fig 5A), of which 28 pathways were enriched significantly with P values less than 0.05 (see Fig 5B and S3 Table). The enriched pathways were mainly involved in carbohydrate metabolism (pyruvate metabolism, glycolysis or gluconeogenesis, synthesis and degradation of ketone bodies), lipid metabolism (fatty acid biosynthesis, glycerophospholipid metabolism, sphingolipid metabolism), amino acid metabolism (phenylalanine metabolism, tyrosine metabolism, valine, leucine, and isoleucine metabolism), and purine metabolism, which play important roles in the rapid growth of cancer tissue. The rapid proliferation of cancer cells requires more ATP as well as nucleotides, proteins, fatty acids, and membrane lipids, which explains the perturbation of metabolites involved in these pathways.

### 3.6. Transcriptomics data analysis and joint analysis

The gene expression profiles of 1027 NSCLC tissues and 108 adjacent tissues obtained from TCGA were analyzed in this study. Genes with statistical significance (2202 genes list in S4 Table) were further analyzed by enrichment pathways analysis based on the Kyoto Encyclopedia of Genes and Genomes (KEGG) database. A total of 293 KEGG pathways were enriched (S5 Table).

### 3.7. Joint analysis of metabolomics and transcriptomics data

Joint analysis of two omics data was conducted to verify each other in the current work. We believed that the combination of metabolomics and transcriptomics analysis in current work would lead to more insight into these fields than either approach alone.

The transcriptomics analysis enables us to validate the metabolism dysregulations revealed by metabolomics work. A total of 27 KEGG pathways were obtained repeatedly from both metabolomics and transcriptomics data-based pathway analysis (Fig 5A). Among the 27 KEGG pathways, 11 were enriched with a significant difference between healthy and NSCLC samples, including glycolysis or gluconeogenesis, tyrosine metabolism, purine metabolism, phenylalanine metabolism, glycine, serine and threonine metabolism, tryptophan metabolism, glycerophospholipid metabolism, nitrogen metabolism, pentose phosphate pathway, and sphingolipid metabolism (S5 Table).

To achieve a better understanding of dysregulated genes and metabolites, we employed a pathway-based approach and integrated different levels of omics in the biological process. Network analysis of comprehensive gene expression and metabolites with noteworthy differences was performed with MetaboAnalyst 4.0 to explore their relationships. Network analysis was conducted using the logarithm of the fold changes in the level of metabolites between the control and NSCLC samples and differentially expressed genes to Metaboanalyst, and a model of the network of metabolites and genes was assembled (see Fig 5C). Genes up-regulated are colored in red, while genes down-regulated are colored in green. Metabolites are represented by blue squares. As can be seen, some metabolites were associated with multiple genes, making the analysis of variation complicated.

### 3.8. Biochemical explanation of the differential metabolites

Nucleotide metabolism imbalance plays an important role in cancers. In this work, the serum levels of inosine and hypoxanthine in NSCLC group fell to less than 10% of those in the control group. Decline of serum inosine or hypoxanthine has been reported in lung cancer [3] and cystic fibrosis lung disease [11].It was inferred that the higher propagation rate of tumor cells led to the decline in serum levels of inosine and hypoxanthine. By transcriptome analysis, 26 genes involved in nucleotide metabolism showed significant differences, with a P-value of 1.72×10^−5^.

Alterations of amino acid metabolism were detected in NSCLC. Serum levels of the aromatic amino acids (tyrosine, tryptophan, and phenylalanine) and branched-chain amino acids (BCAA, including valine, leucine, and isoleucine) were previously reported to decrease in lung cancer [7, 12]. In consistent, our research has revealed a 20–70% decrease in serum levels of aromatic amino acids and BCAAs in NSCLC patients. The reduction would be due both to the malnutrition associated with the tumor-bearing state and to an increase of the amino acid demand in tumor [12, 13]. Transcriptomics analysis revealed that levels of mRNAs for enzymes of amino acid metabolism were significantly altered, verifying alterations of amino acid metabolism in NSCLC group.

Taurine and hypotaurine metabolism has been shown to be relevant to lung cancer [14]. Consistent with previous report [14], decreased taurine level was noticed in NSCLC group in current research. However, taurine and hypotaurine metabolism was not detected significantly altered by transcriptomics analysis. The inconsistence may be explained by that selective information was provides by tissue samples while whole body information was carried out in serum sample.

Disturbed permeability of cellular membrane was noticed in the current research. Low level indoleacrylic acid and significant variations in Phosphatidylethanolamine (PE) and Phosphatidylcholines (PC) levels could provide evidence of disturbed permeability of cellular membrane [15]. In our research, the serum level of indoleacrylic acid was decreased in NSCLC patients, indicating disturbed cell membrane permeability [16]. Consistent with pervious report [17–20], our results revealed elevation of lysoPE(16:0), lysoPE(20:4), lysoPE(18:0), lysoPE(22:6) and decline of PC(18:0), LysoPC (18:2), LysoPC(18:1), LysoPC(18:0), LysoPC (16:0), as well as LysoPC(17:0) in NSCLC patients, suggesting aberrations in lipid metabolism. Transcriptomics data verified metabolomics results and showed corresponding changes in the messenger RNAs of relevant enzymes, with roles in lipolysis in adipocytes, glycerophospholipid metabolism, and glycerolipid metabolism.

Dysregulated sphingolipid metabolism appears to occur frequently in human cancers [21]. Metabolites of sphingolipids (phytosphingosine and sphingosine) are known to influence numerous cellular functions [22]. Serum levels of sphingolipids were reported to increase in ovarian [23] and endometrial cancer [24]. Our work revealed that the serum levels of phytosphingosine, C16 sphinganine, and sphingosine were elevated in NSCLC, indicating that sphingolipid metabolism has an important role in NSCLC. Consistently, significantly altered sphingolipid metabolism was revealed by transcriptomics analysis, with a P-value less than 0.05.

Linoleic acid is a vital component of cell membranes and is also the precursor of arachidonic acid, which is highly active in the proinflammatory response [25]. Levels of linoleic acid and arachidic acid (arachidate) were reported to be higher in cancer tissue [7, 26]. Platelet linoleic acid has been reported as a biomarker for advanced NSCLC [27]. In line with previous research, we observed an increase in the serum levels of linoleic acid and arachidic acid in NSCLC patients.

Oleamide has been considered as a new class of biological signaling molecule because of its role in cancer [28, 29]. Oleamide levels were found to decrease in lung cancer [4, 7]. Stearic acid, oxidative product of oleic acid, was reported to significantly increased in tumor tissues [6, 7]. In agreement with previous reports, our research has revealed an elevation of serum stearic acid and a decline of serum oleamide in NSCLC patients.

Balance between fatty acid synthesis and *β*-oxidation were altered in tumor metabolic adaptation [30, 31]. Decanoic acid (capric acid) was reported to reduce cancer cell viability in vitro [32], and increased more than 10-fold in colorectal cancer [33]. In present study, serum level of decanoic acid was increased by 23 folds in the NSCLC group. Transcriptomics analysis supported the alteration, with eight genes downregulated leading to an elevation of decanoic acid (Fig 5C). Carnitine plays a key role in fatty acid *β*-oxidation. Researches have reported decreased serum acylcarnitine concentrations in cancer patients [34]. In current research, serum acyl-carnitine C10:1 was found significantly decreased to 35% in NSCLC group, suggesting altered fatty acid *β*-oxidation. However, fatty acid *β*-oxidation was not revealed significantly altered by transcriptomics analysis. This may be derived from tissue discrepancy between metabolomics analysis and transcriptomics analysis.

Regulation of glucose metabolism in carcinogenesis is a multi-factor, multi-step process. Higher serum glucose levels were reported in patients with NSCLC [35], suggesting upregulated gluconeogenesis. Level of serum lactate was reported to decline significantly in lung cancer patients compared with healthy controls [7]. In our research, a 40% elevation of serum glucose and a 30% decrease in lactate were noticed, indicating altered gluconeogenesis and glycolysis. We further verified the alteration by transcriptomics analysis, revealing significant disruption of glycolysis or gluconeogenesis (16 genes involved, P-value 4.55×10^−6^).

Overall, in the current research, both metabolomics and transcriptomics analysis revealed disturbances in nucleotide and amino acid metabolism, lysophospholipid (lysoPCs) catabolism, glycerophospholipid metabolism, fatty acid synthase and fatty acid metabolism, sphingolipid metabolism, gluconeogenesis, and glycolysis in NSCLC patients.

### 3.9. Limitation of the work

The current work was limited by the following factors. First, metabolomics and transcriptomics data were collected from different populations; second, metabolomics analysis were based on serum samples, while TCGA transcriptomics data were of cancerous or adjacent peritumoral tissues; third, healthy volunteers and NSCLC groups were not enrolled with matched ages. These may lead to inconformity of the metabolic information. However, the deficiencies may be eliminated by relatively big sample size in current work; sample heterogeneities were minimized the during sample collection, storage, and preparation. Besides, serum carries information from entire body while cancer tissues only provided selective cancerous information, causing partial inconsistence between metabolomics and transcriptomics work. As a result, sensitivity analysis was conducted by multivariable analysis plotting NSCLC group, where NSCLC patients were grouped according to ages (group1 with ages 47.07±7.15, group2 with ages 64.49±6.34). It turned out that no obvious distinction was noticed between group 1 and group 2 (S2 Fig). Thus, integrated analysis of metabolomics and transcriptomics in current work verified each other and provided convincing metabolic information of NSCLC.

## 4. Conclusions

We performed an integrated analysis of metabolomics and transcriptomics to explore metabolism characteristics in NSCLC. In total, 35 differential metabolites and 2202 genes with significantly difference were defined. Metabolic disturbances revealed in metabolomics analysis were further verified by transcriptomics analysis. A combinational biomarkers of hypoxanthine, inosine, L-tryptophan, indoleacrylic acid, acyl-carnitine C10:1, and lysoPC(18:2) was established as a promising method for NSCLC diagnosis and screening. Finally, we built a fully connected network of metabolites and genes, which shows a good correspondence between the transcriptome analysis and the metabolites selected for diagnosis. This work demonstrated that integration of metabolomics and transcriptomics data was a promising method to investigate the mechanism of carcinogenesis and discover more reliable biomarkers.

## Supporting information

S1 Checklist (DOCX)

S1 Fig The PCA score plots of the patients with NSCLC and the healthy controls showed clear separation in both positive and negative mode. (JPG)

S2 Fig The PCA score plots of the patients with NSCLC (group 1 and group 2) and the healthy controls for sensitivity analysis of ages. (JPG)

S1 Table Drift of retention times, m/z and the RSD of peak areas of 6 selected characteristic features from QC samples during the analysis. (DOCX)

S2 Table List of significant metabolites of serum samples from NSCLC patients compared with those of healthy people examined in both ESI+ and ESI- modes. (DOCX)

S3 Table Pathway analysis of metabolites identified by metabolomics analysis. The table below shows the detailed results from the pathway analysis. The Total is the total number of compounds in the pathway; the Hits is the number actually matched from the user uploaded data; the Raw p is the original P-value calculated from the enrichment analysis; the Holm p is the P-value adjusted by the Holm-Bonferroni method; the FDR p is the P-value adjusted using the False Discovery Rate; and the Impact is the pathway impact value calculated from pathway topology analysis.
(DOCX)

S4 Table Genes significantly altered in NSCLC as detected by transcriptomics analysis. (XLSX)

S5 Table Part of overlapping KEGG pathways enriched by transcriptomics analysis. It was revealed by transcriptomics analysis that 11 of the 27 overlapping KEGG pathways were significantly altered, while by metabolomics analysis, 27 had significant differences.
(DOC)

S6 Table R2X, R2Y and Q2 of multivariable analysis in ESI+ or ESI- mode by PCA-X or OPLS-DA. (DOCX)
