# Supplementary figures and images for: A comprehensive analysis of metabolomics and transcriptomics in non-small cell lung cancer
Source: PLoS One. 2020 May 6;15(5):e0232272. doi: 10.1371/journal.pone.0232272 (PMC7202610; doi:10.1371/journal.pone.0232272)

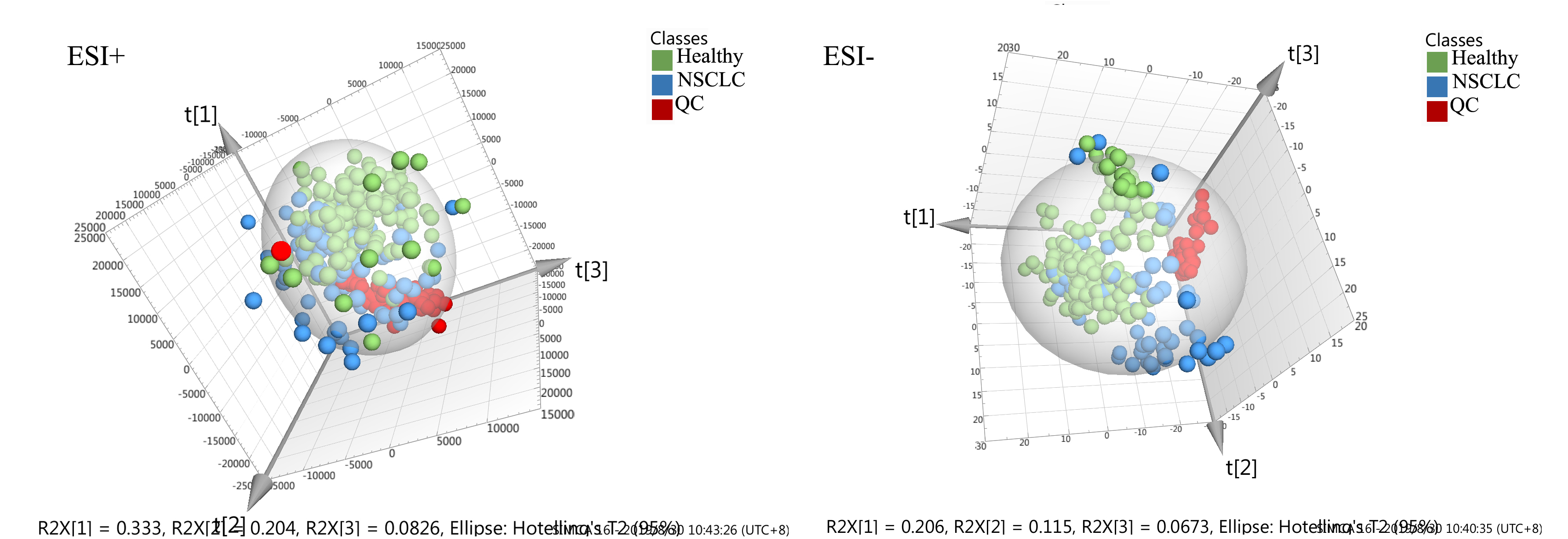

Supplement: S1 Fig — (JPG) [file pone.0232272.s002.jpg]

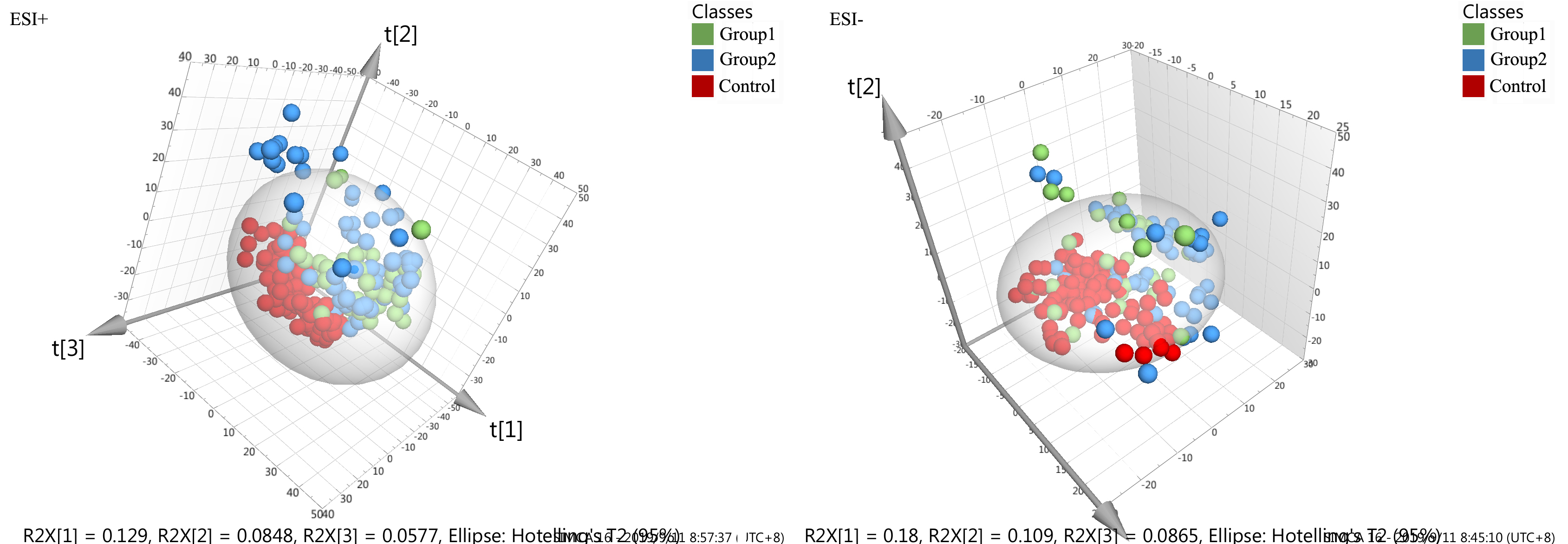

Supplement: S2 Fig — (JPG) [file pone.0232272.s003.jpg]
