# Supplementary material for: A comprehensive analysis of metabolomics and transcriptomics in non-small cell lung cancer
Source: PLoS One. 2020 May 6;15(5):e0232272. doi: 10.1371/journal.pone.0232272 (PMC7202610; doi:10.1371/journal.pone.0232272)
Supplement: S1 Table — (DOCX) [file pone.0232272.s004.docx]

Table S1 RSD of peak areas of 6 selected characteristic features from QC samples during the analysis in ESI+ or ESI- mode.

| Mode | ESI+ | ESI+ | ESI+ | ESI- | ESI- | ESI- |
| --- | --- | --- | --- | --- | --- | --- |
| Mass features | 122.0576_1.72 | 332.2438_2.83 | 810.6034_6.18 | 107.0504_2.31 | 498.2880_3.40 | 738.9993_0.74 |
| RSD of peak area | 13.37 | 9.29 | 13.97 | 14.43 | 12.98 | 13.80 |
