# Supplementary material for: A comprehensive analysis of metabolomics and transcriptomics in non-small cell lung cancer
Source: PLoS One. 2020 May 6;15(5):e0232272. doi: 10.1371/journal.pone.0232272 (PMC7202610; doi:10.1371/journal.pone.0232272)
Supplement: S2 Table — (DOCX) [file pone.0232272.s005.docx]

Table S2 List of significant metabolites of serum samples for NSCLC patients compared with healthy people in both ESI+ and ESI- modes.

| **No.** | **Mode** | **tR**  **(min)** | **m/z** | **Mass error**  **(ppm)** | **Metabolites** | **HMDB/METLIN** | **Formula** | **Adduct** | **Fold change** | **p value** | **VIP** | **Pathway involved** |
| --- | --- | --- | --- | --- | --- | --- | --- | --- | --- | --- | --- | --- |
| 1 | ESI- | 0.64 | 124.0077 | 0.80 | Taurine | HMDB0000251 | C_2_H_7_NO_3_S | [M-H]^1-^ | 0.52↓* | 1.12E-12 | 1.80 | Taurine and hypotaurine metabolism |
| 2 | ESI- | 0.65 | 89.0237 | -11.11 | Lactic acid | HMDB0000190 | C_3_H_6_O_3_ | [M-H]^1-^ | 0.68↓* | 4.96E-13 | 1.94 | Glycolysis or Gluconeogenesis |
| 3 | ESI+ | 0.68 | 203.052 | -6.66 | Glucose | HMDB0000122 | C_6_H_12_O_6_ | [M+Na]^1+^ | 1.39 ↑* | 6.79E-20 | 3.95 | Glycolysis or Gluconeogenesis |
| 4 | ESI- | 0.7 | 103.0405 | 0.96 | 3-Hydroxybutyric acid | HMDB0000357 | C_4_H_8_O_3_ | [M-H]^1-^ | 2.17 ↑* | 1.64E-22 | 2.24 | Synthesis and degradation of ketone bodies |
| 5 | ESI- | 1.04 | 112.052 | 0.88 | Creatinine | HMDB0000562 | C_4_H_7_N_3_O | [M-H]^1-^ | 0.82↓* | 2.20E-06 | 1.35 | Arginine and proline metabolism |
| 6 | ESI+ | 1.38 | 137.0451 | -2.94 | Hypoxanthine | HMDB0000157 | C_5_H_4_N_4_O | [M+H]^1+^ | 0.16↓* | 9.10E-43 | 2.70 | [Purine Metabolism](http://www.hmdb.ca/metabolite_ontology_terms/3322298) |
| 6 | ESI- | 1.40 | 135.0308 | -5.15 | Hypoxanthine | HMDB0000157 | C_5_H_4_N_4_O | [M-H]^1-^ | 0.11↓* | 1.29E-38 | 2.90 | Purine Metabolism |
| 7 | ESI- | 1.39 | 180.0663 | -3.31 | L-Tyrosine | HMDB0000158 | C_9_H_11_NO_3_ | [M-H]^1-^ | 0.79↓* | 4.91E-08 | 1.63 | Tryptophan metabolism |
| 8 | ESI+ | 1.47 | 291.0699 | -2.61 | Inosine | HMDB0000195 | C_10_H_12_N_4_O_5_ | [M+Na]^1+^ | 0.09↓* | 4.18E-38 | 2.15 | Purine Metabolism |
| 8 | ESI+ | 1.49 | 269.0883 | 1.86 | Inosine | HMDB0000195 | C_10_H_12_N_4_O_5_ | [M+H]^1+^ | 0.05↓* | 8.27E-27 | 1.25 | Purine Metabolism |
| 8 | ESI- | 1.53 | 267.0726 | -4.48 | Inosine | HMDB0000195 | C_10_H_12_N_4_O_5_ | [M-H]^1-^ | 0.07↓* | 9.23E-21 | 2.23 | Purine Metabolism |
| 9 | ESI- | 1.74 | 164.0717 | -1.82 | Phenylalanine | HMDB0000159 | C_9_H_11_NO_2_ | [M-H]^1-^ | 0.84↓* | 1.01E-06 | 1.46 | Phenylalanine metabolism |
| 10 | ESI+ | 1.96 | 188.0697 | -3.21 | Indoleacrylic acid | HMDB0000734 | C_11_H_9_NO_2_ | [M+H]^1+^ | 0.66↓* | 1.07E-37 | 2.51 | Indoleacrylic acid metabolism |
| 11 | ESI+ | 1.97 | 205.0962 | 0.98 | L-Tryptophan | HMDB0000929 | C_11_H_12_N_2_O_2_ | [M+H]^1+^ | 0.64↓* | 9.76E-42 | 2.31 | [Tryptophan Metabolism](http://www.hmdb.ca/metabolite_ontology_terms/3325783) |
| 11 | ESI- | 1.95 | 203.0822 | -3.43 | L-Tryptophan | HMDB0000929 | C_11_H_12_N_2_O_2_ | [M-H]^1-^ | 0.53↓* | 1.86E-37 | 3.06 | Tryptophan metabolism |
| 12 | ESI+ | 3.16 | 314.2321 | -0.64 | acyl-carnitine C10:1 | HMDB0013205 | C_17_H_31_NO_4_ | [M+H]^1+^ | 0.35↓* | 7.71E-42 | 2.89 | Lipid metabolism |
| 13 | ESI+ | 3.55 | 274.2731 | -2.56 | C16-Sphinganine |  | C_16_H_35_NO_2_ | [M+H]^1+^ | 1.20↑* | 1.75E-04 | 3.42 | sphingolipid metabolism pathway |
| 14 | ESI+ | 3.57 | 318.2995 | -1.58 | Phytosphingosine | HMDB0004610 | C_18_H**_39_**NO_3_ | [M+H]^1+^ | 1.24↑* | 8.01E-05 | 2.88 | Sphingolipid metabolism pathway |
| 15 | ESI+ | 4.03 | 302.3049 | -0.66 | Sphinganine | HMDB0000269 | C_18_H_39_NO_2_ | [M+H]^1+^ | 1.34↑* | 1.61E-06 | 2.76 | Sphingolipid metabolism pathway |
| 16 | ESI- | 4.07 | 171.1384 | -5.23 | Capric acid | HMDB0000511 | C_10_H_20_O_2_ | [M-H]^1-^ | 22.91↑* | 6.03E-19 | 2.11 | Fatty acid biosynthesis |
| 17 | ESI+ | 4.73 | 330.3361 | -3.52 | Arachidate | HMDB0002212 | C_20_H_40_O_2_ | [M+NH4]^1+^ | 1.33↑* | 4.79E-05 | 2.69 | Fatty acid metabolism |
| 18 | ESI+ | 5.01 | 494.3242 | 0.81 | LysoPC(16:1) | HMDB0010383 | C_24_H_48_NO_7_P | [M+H]^1+^ | 1.00 | 8.70E-01 | 1.69 | Lipid metabolism |
| 19 | ESI+ | 5.31 | 526.2932 | 1.14 | LysoPE(22:6) | HMDB0011526 | C_27_H_44_NO_7_P | [M+H]^1+^ | 1.15↑* | 3.59E-04 | 1.28 | Lipid metabolism |
| 20 | ESI+ | 5.35 | 502.2935 | 1.79 | LysoPE(20:4) | HMDB0011517 | C_25_H_44_NO_7_P | [M+H]^1+^ | 1.23↑* | 1.81E-09 | 2.12 | Lipid metabolism |
| 21 | ESI+ | 5.43 | 520.3799 | -7.11 | LysoPC(18:2) | HMDB0010386 | C_27_H_55_NO_6_P | [M+H]^1+^ | 0.72↓* | 1.01E-26 | 14.47 | Lysophospholipid catabolism |
| 22 | ESI- | 5.88 | 583.2569 | 0.68 | Bilirubin | HMDB0000054 | C_33_H_36_N_4_O_6_ | [M-H]^1-^ | 0.63↓* | 2.91E-10 | 1.47 | Primary bile acid biosynthesis |
| 23 | ESI+ | 5.76 | 454.293 | 0.88 | LysoPE(16:0) | HMDB0011503 | C_21_H_44_NO_7_P | [M+H]^1+^ | 1.49↑* | 8.07E-12 | 1.84 | Lipid metabolism |
| 24 | ESI+ | 5.92 | 496.3392 | -0.61 | LysoPC(16:0) | HMDB0010382 | C_24_H_50_NO_7_P | [M+H]^1+^ | 0.92↓* | 2.65E-05 | 11.71 | Lysophospholipid catabolism |
| 25 | ESI+ | 6.32 | 522.3560 | 1.73 | LysoPC(18:1) | HMDB0010385 | C_26_H_52_NO_7_P | [M+H]^1+^ | 0.91↓* | 7.06E-04 | 6.19 | Glycerophospholipid metabolism |
| 26 | ESI- | 6.9 | 480.3095 | -0.62 | LysoPE(18:0) | HMDB0011130 | C_23_H_48_NO_7_P | [M-H]^1-^ | 0.78↓* | 1.82E-08 | 1.86 | Glycerophospholipid metabolism |
| 27 | ESI+ | 7.37 | 482.3242 | 0.83 | LysoPE(18:0) | HMDB0011130 | C_23_H_48_NO_7_P | [M+H]^1+^ | 1.41↑* | 6.46E-06 | 1.66 | Lipid metabolism |
| 28 | ESI+ | 7.59 | 524.3716 | 1.53 | LysoPC(18:0) | HMDB0010384 | C_26_H_54_NO_7_P | [M+H]^1+^ | 0.90↓* | 2.53E-05 | 8.60 | Lysophospholipid catabolism |
| 29 | ESI- | 8.75 | 508.3414 | 0.59 | LysoPC(17 : 0) | HMDB0012108 | C_25_H_52_NO_7_P | [M-H]^1-^ | 0.64↓* | 3.74E-13 | 1.98 | Lipid metabolism |
| 30 | ESI+ | 8.78 | 279.2318 | 0.72 | Alpha-Linolenic acid | HMDB0001388 | C_18_H_30_O_2_ | [M+H]^1+^ | 1.41↑* | 3.27E-07 | 2.04 | Lipid metabolism |
| 31 | ESI+ | 9.63 | 282.2787 | -0.71 | Oleamide | HMDB0002117 | C_18_H_35_NO | [M+H]^1+^ | 0.90↓ | 1.06E-02 | 6.11 | Lipid metabolism |
| 32 | ESI+ | 9.75 | 281.2472 | -5.35 | linoleate | HMDB0000673 | C_18_H_32_O_2_ | [M+H]^1+^ | 1.28↑* | 1.92E-06 | 2.72 | Linoleic acid metabolism |
| 32 | ESI+ | 9.76 | 263.2373 | 2.5 | linoleate | HMDB0000673 | C_18_H_32_O_2_ | [M-H2O+H]^1+^ | 1.26↑* | 1.53E-05 | 1.67 | Linoleic acid metabolism |
| 33 | ESI+ | 9.78 | 780.5537 | 0.38 | PC(36:5) |  | C_44_H_78_NO_8_P | [M+H]^1+^ | 1.09↑ | 7.74E-02 | 2.68 | Lipid metabolism |
| 34 | ESI- | 10.96 | 281.2463 | -9.21 | Oleic acid | HMDB0000573 | C_18_H_34_O_2_ | [M-H]^1-^ | 1.18↑* | 1.53E-05 | 1.05 | Fatty acid biosynthesis |
| 35 | ESI- | 11 | 283.2649 | 1.41 | Stearic acid | HMDB0000827 | C_18_H_36_O_2_ | [M-H]^1-^ | 1.22↑* | 3.94E-06 | 1.13 | Fatty acid biosynthesis |

Note: serum metabolites up-regulated in NSCLC patients compared with healthy people were indicated by “↑”; metabolites down-regulated was indicated by “↓”; fold changes with significant difference (p <0.05) were indicated by “*”.
