# Supplementary material for: A comprehensive analysis of metabolomics and transcriptomics in non-small cell lung cancer
Source: PLoS One. 2020 May 6;15(5):e0232272. doi: 10.1371/journal.pone.0232272 (PMC7202610; doi:10.1371/journal.pone.0232272)
Supplement: S3 Table — The table below shows the detailed results from the pathway analysis. The Total is the total number of compounds in the pathway; the Hits is the number actually matched from the user uploaded data; the Raw p is the original P-value calculated from the enrichment analysis; the Holm p is the P-value adjusted by the Holm-Bonferroni method; the FDR p is the P-value adjusted using the False Discovery Rate; and the Impact is the pathway impact value calculated from pathway topology analysis. (DOCX) [file pone.0232272.s006.docx]

Table S3 Pathway analysis of metabolitesidentified by metabolomics analysis.

| KEGG metabolic pathways | Total Cmpd | Hits | Raw p | -log(p) | Holm adjust | FDR | Impact |
| --- | --- | --- | --- | --- | --- | --- | --- |
| Valine, leucine and isoleucine biosynthesis | 27 | 3 | 5.91E-58 | 1.32E+02 | 1.71E-56 | 8.56E-57 | 0.04 |
| Valine, leucine and isoleucinedegradation | 40 | 3 | 5.91E-58 | 1.32E+02 | 1.71E-56 | 8.56E-57 | 0.02 |
| Tryptophan metabolism | 79 | 1 | 1.85E-37 | 8.46E+01 | 4.99E-36 | 1.34E-36 | 0.11 |
| Glycine, serine and threonine metabolism | 48 | 1 | 1.85E-37 | 8.46E+01 | 4.99E-36 | 1.34E-36 | 0.00 |
| Propanoate metabolism | 35 | 2 | 1.90E-36 | 8.22E+01 | 4.76E-35 | 1.10E-35 | 0.00 |
| Purine metabolism | 92 | 2 | 5.00E-30 | 6.75E+01 | 1.20E-28 | 2.42E-29 | 0.01 |
| Nitrogen metabolism | 39 | 4 | 4.62E-26 | 5.83E+01 | 1.06E-24 | 1.91E-25 | 0.00 |
| Phenylalanine, tyrosine and tryptophanbiosynthesis | 27 | 3 | 1.01E-25 | 5.76E+01 | 2.21E-24 | 3.64E-25 | 0.01 |
| Pantothenate and CoA biosynthesis | 27 | 1 | 1.82E-25 | 5.70E+01 | 3.81E-24 | 5.85E-25 | 0.00 |
| Butanoate metabolism | 40 | 1 | 1.67E-22 | 5.01E+01 | 3.35E-21 | 4.41E-22 | 0.00 |
| Synthesis and degradation of ketone bodies | 6 | 1 | 1.67E-22 | 5.01E+01 | 3.35E-21 | 4.41E-22 | 0.00 |
| Aminoacyl-tRNA biosynthesis | 75 | 7 | 4.03E-22 | 4.93E+01 | 7.26E-21 | 9.74E-22 | 0.00 |
| Glycolysis or Gluconeogenesis | 31 | 2 | 4.05E-21 | 4.70E+01 | 6.88E-20 | 9.02E-21 | 0.00 |
| Starch and sucrose metabolism | 50 | 1 | 6.79E-20 | 4.41E+01 | 1.09E-18 | 1.16E-19 | 0.02 |
| Galactose metabolism | 41 | 1 | 6.79E-20 | 4.41E+01 | 1.09E-18 | 1.16E-19 | 0.00 |
| Pentose phosphate pathway | 32 | 1 | 6.79E-20 | 4.41E+01 | 1.09E-18 | 1.16E-19 | 0.00 |
| Amino sugar and nucleotide sugarmetabolism | 88 | 1 | 6.79E-20 | 4.41E+01 | 1.09E-18 | 1.16E-19 | 0.00 |
| Glycerophospholipid metabolism | 39 | 1 | 3.75E-13 | 2.86E+01 | 4.50E-12 | 6.04E-13 | 0.00 |
| Pyruvate metabolism | 32 | 1 | 4.95E-13 | 2.83E+01 | 5.45E-12 | 7.56E-13 | 0.14 |
| Taurine and hypotaurine metabolism | 20 | 1 | 1.11E-12 | 2.75E+01 | 1.11E-11 | 1.62E-12 | 0.33 |
| Porphyrin and chlorophyll metabolism | 104 | 1 | 2.91E-10 | 2.20E+01 | 2.62E-09 | 4.02E-10 | 0.01 |
| Tyrosine metabolism | 76 | 1 | 4.90E-08 | 1.68E+01 | 3.92E-07 | 5.92E-08 | 0.05 |
| Ubiquinone and other terpenoid-quinonebiosynthesis | 36 | 1 | 4.90E-08 | 1.68E+01 | 3.92E-07 | 5.92E-08 | 0.00 |
| Thiamine metabolism | 24 | 1 | 4.90E-08 | 1.68E+01 | 3.92E-07 | 5.92E-08 | 0.00 |
| Phenylalanine metabolism | 45 | 2 | 8.47E-08 | 1.63E+01 | 4.23E-07 | 9.82E-08 | 0.12 |
| Fatty acid biosynthesis | 49 | 4 | 3.54E-06 | 1.26E+01 | 1.41E-05 | 3.94E-06 | 0.00 |
| Sphingolipid metabolism | 25 | 2 | 1.26E-05 | 1.13E+01 | 3.79E-05 | 1.36E-05 | 0.14 |
| Arginine and proline metabolism | 77 | 2 | 5.61E-03 | 5.18E+00 | 1.12E-02 | 5.81E-03 | 0.11 |
| PPrimary bile acidbiosynthesis | 47 | 2 | 6.28E-02 | 2.77E+00 | 6.28E-02 | 6.28E-02 | 0.02 |

The table below shows the detailed results from the pathway analysis. The Total is the total number of compounds in the pathway; the Hits is the actually matched number from the user uploaded data; the Raw p is the original p value calculated from the enrichment analysis; the Holm p is the p value adjusted by Holm-Bonferroni method; the FDR p is the p value adjusted using False Discovery Rate; the Impact is the pathway impact value calculated from pathway topology analysis.
