# Supplementary material for: A comprehensive analysis of metabolomics and transcriptomics in non-small cell lung cancer
Source: PLoS One. 2020 May 6;15(5):e0232272. doi: 10.1371/journal.pone.0232272 (PMC7202610; doi:10.1371/journal.pone.0232272)
Supplement: S5 Table — It was revealed by transcriptomics analysis that 11 of the 27 overlapping KEGG pathways were significantly altered, while by metabolomics analysis, 27 had significant differences. (DOC) [file pone.0232272.s008.doc]

Table S5 Part of overlapped KEGG pathways enriched by transcriptomics analysis.

| KEGG PATHWAY | ID | Input number | Background number | P-Value | Corrected P-Value | Input |
| --- | --- | --- | --- | --- | --- | --- |
| Glycolysis or Gluconeogenesis | hsa00010 | 16 | 67 | 4.55E-06 | 2.72E-05 | ALDH2/GAPDH/LDHA/PFKP/TPI1/ALDH3A1/HK3/GPI/ADH7/FBP1/ADH1B/ENO1/ALDOA/ALDH3B2/ALDH3B1/PKM |
| Tyrosine metabolism | hsa00350 | 11 | 35 | 1.67E-05 | 7.87E-05 | MIF/MAOB/MAOA/AOC3/AOX1/ALDH3A1/ADH7/ADH1B/IL4I1/ALDH3B2/ALDH3B1 |
| Purine metabolism | hsa00230 | 26 | 176 | 1.72E-05 | 8.00E-05 | PDE2A/ADCY9/ENPP4/PPAT/XDH/POLR2H/HPRT1/PKM/PAICS/RRM1/RRM2/PDE5A/PAPSS2/AK1/AK4/NPR1/ADA/GUCY1A2/NME2/NME1/NME4/ADCY4/POLD2/ATIC/POLE2/GMPS |
| Phenylalanine metabolism | hsa00360 | 8 | 17 | 2.42E-05 | 0.000109 | MIF/MAOB/MAOA/AOC3/ALDH3A1/IL4I1/ALDH3B2/ALDH3B1 |
| Glycine, serine and threonine metabolism | hsa00260 | 9 | 40 | 0.00079872 | 0.002294 | MAOB/PSAT1/GCAT/AOC3/GAMT/PSPH/MAOA/SHMT2/PHGDH |
| Arginine and proline metabolism | hsa00330 | 10 | 50 | 0.00092207 | 0.002573 | ALDH2/MAOB/MAOA/ALDH18A1/GAMT/PYCR1/AOC1/SMOX/SRM/CKMT1B |
| Tryptophan metabolism | hsa00380 | 8 | 40 | 0.00293534 | 0.006772 | CAT/ALDH2/MAOB/MAOA/INMT/AOX1/AOC1/IL4I1 |
| Glycerophospholipid metabolism | hsa00564 | 13 | 95 | 0.00374878 | 0.008449 | PLA2G1B/PLA2G16/PLPP3/LPCAT1/GPD1/SELENOI/DGKA/GPD1L/PLA2G2A/PLPP2/PLA2G4F/ETNK2/PNPLA6 |
| Nitrogen metabolism | hsa00910 | 5 | 17 | 0.00456842 | 0.009989 | CA4/CA9/CA3/CA12/CA2 |
| Pentose phosphate pathway | hsa00030 | 6 | 29 | 0.00834602 | 0.016523 | FBP1/RGN/PFKP/G6PD/ALDOA/GPI |
| Sphingolipid metabolism | hsa00600 | 7 | 47 | 0.02036504 | 0.037062 | UGT8/SGPL1/CERS3/SGMS2/PLPP3/PLPP2/ASAH1 |
| Amino sugar and nucleotide sugar metabolism | hsa00520 | 6 | 48 | 0.0585253 | 0.093195 | TSTA3/HK3/CHIA/UGDH/GNPNAT1/GPI |
| Galactose metabolism | hsa00052 | 4 | 31 | 0.10411653 | 0.152531 | AKR1B10/PFKP/HK3/B4GALT2 |
| Starch and sucrose metabolism | hsa00500 | 6 | 57 | 0.10526685 | 0.153449 | KL/HK3/PGM2L1/UGDH/UGT1A6/GPI |
| Pyruvate metabolism | hsa00620 | 4 | 40 | 0.19012419 | 0.253211 | PC/LDHA/PKM/ALDH2 |
| Porphyrin and chlorophyll metabolism | hsa00860 | 4 | 42 | 0.21165954 | 0.27789 | CPOX/UGT1A6/CP/HMBS |
| Primary bile acid biosynthesis | hsa00120 | 2 | 17 | 0.25638045 | 0.322401 | HSD17B4/CYP27A1 |
| Phenylalanine, tyrosine and tryptophan biosynthesis | hsa00400 | 1 | 5 | 0.27208611 | 0.336377 | IL4I1 |
| Fatty acid metabolism | hsa01212 | 4 | 48 | 0.27963418 | 0.342815 | ACADL/ACSL4/HACD4/ACSL1 |
| Synthesis and degradation of ketone bodies | hsa00072 | 1 | 10 | 0.44136109 | 0.515214 | BDH1 |
| Ubiquinone and other terpenoid-quinone biosynthesis | hsa00130 | 1 | 11 | 0.47016611 | 0.531887 | NQO1 |
| Taurine and hypotaurine metabolism | hsa00430 | 1 | 11 | 0.47016611 | 0.531887 | CDO1 |
| Aminoacyl-tRNA biosynthesis | hsa00970 | 4 | 66 | 0.49009386 | 0.550182 | GARS/VARS/DARS2/TARS |
| Valine, leucine and isoleucine degradation | hsa00280 | 3 | 48 | 0.49306927 | 0.550992 | ALDH2/IL4I1/AOX1 |
| Propanoate metabolism | hsa00640 | 2 | 32 | 0.52907726 | 0.576281 | ACSS3/LDHA |
| Pantothenate and CoA biosynthesis | hsa00770 | 1 | 18 | 0.63425047 | 0.668473 | DPYD |
| Butanoate metabolism | hsa00650 | 1 | 28 | 0.78462326 | 0.801027 | BDH1 |

Note: It was revealed by transcriptomics analysis that 11 of the 27 overlapped KEGG pathways were with significance alterations, While by metabolomics analysis 27 were with significance differences.
