# Supplementary material for: A comprehensive analysis of metabolomics and transcriptomics in non-small cell lung cancer
Source: PLoS One. 2020 May 6;15(5):e0232272. doi: 10.1371/journal.pone.0232272 (PMC7202610; doi:10.1371/journal.pone.0232272)
Supplement: S6 Table — (DOCX) [file pone.0232272.s009.docx]

Table S6: R2X, R2Y and Q2 of multivariable analysis in ESI+ or ESI- mode by PCA-X or OPLS-DA.

| Model | R2X | R2Y | Q2 |
| --- | --- | --- | --- |
| PCA (ESI+) | 0.691 | -- | 0.356 |
| PCA (ESI-) | 0.684 | -- | 0.429 |
| OPLS-DA (ESI+) | 0.370 | 0.915 | 0.855 |
| OPLS-DA(ESI-) | 0.369 | 0.904 | 0.816 |
